# Comparative metagenomic characterization of gut microbiota and antibiotic resistome in multi-facility SPF mice

**DOI:** 10.1186/s12866-025-04699-6

**Published:** 2026-01-15

**Authors:** Yujie Wang, Caihong Wu, Qi Zhu, Chun Fan, Yingying Zhu, Yifei Chen, Xiaofeng Wei, Liping Feng

**Affiliations:** Shanghai Laboratory Animal Research Center, Shanghai, 201203 China

**Keywords:** Metagenomic sequencing, Mice, Gut microbiota, Resistance genes

## Abstract

Specific pathogen-free (SPF) mice are pivotal preclinical models linking basic microbiology to clinical translation, yet comprehensive high-resolution profiling of their gut microbiome, especially antibiotic resistance genes (ARGs), remains limited. To address this gap, metagenomic sequencing was conducted on cecal contents from C57BL/6 and BALB/c SPF mice from five Shanghai laboratory animal facilities, generating 141 Gbp high-quality sequencing data. From 1,761,909 predicted genes, 1,048,575 non-redundant genes were identified for analysis. Taxonomic annotation identified Bacillota (73.0%), Bacteroidota (16.6%), and Actinomycetota (2.9%) as dominant phyla. At the genus level, microbial communities varied markedly across facilities, with *Muribaculaceae* prevailing in SHA/SHD and *Blautia* or *Enterococcus* enriched in SHB/SHE. Beta diversity analysis showed communities clustered by facility, indicating breeding environment had a stronger impact on gut microbiota diversity than host strain. KEGG, COG, and GO functional annotation revealed broad metabolic and molecular diversity. Antibiotic resistome profiling identified 11 ARG categories, predominantly associated with glycopeptides (18.1%) and tetracycline (11.3%) resistance. The most enriched ARG carriers were Pseudomonadota (*acrD*, *emrB*, *mdtB* etc.), Bacillota (*tet*(44), *tet(M)*, *tet*(O) etc.), Bacteroidota (*tet*(Q), *mel*, *tet(X)* etc.), and Actinomycetota (*rpoB*, *ileS*). Furthermore, ARGs resistance mechanisms varied between facilities with distinct beta-diversity clustering: SHB and SHE mice mainly employed *antibiotic target alteration* against glycopeptides, whereas SHA, SHD, and SHC-C57BL/6 primarily utilized antibiotic target protection against tetracyclines. This study presents a high-resolution comparison of gut microbiota and ARGs in SPF mice from multiple facilities, highlighting facility-dependent microbial and resistome variation and providing valuable references for preclinical microbiological standardization and risk assessment.

**Supplementary Information:**

The online version contains supplementary material available at 10.1186/s12866-025-04699-6.

## Importance

Given the critical role of the gut microbiome in human health, specific pathogen-free (SPF) mice have been internationally recognized as standard laboratory animals for microbiome research, since they are free of pathogens that harm animal health or interfere with experimental outcomes, and only exclude non-interfering pathogens permitted in conventional ordinary mice [1]. SPF mice are the most widely used preclinical models in biomedical research, serving as a critical link between basic microbial mechanism exploration and clinical translation [2]. The gut microbiota of laboratory animals, as a key component of their biological background, plays pivotal roles in regulating host metabolism, immune development, and physiological homeostasis [3]. Concurrently, gut microbiota-associated ARGs have emerged as a non-negligible factor affecting the reliability of preclinical studies, as their carriage and transmission may interfere with the evaluation of drug efficacy and safety [4].

Similar to humans, the murine gut harbors a complex and diverse microbiota that contributes crucially to host physiology and metabolism, whose compositional alterations are closely associated with murine models of human diseases [5]. The compositional plasticity of the murine gut microbiota is modulated by host-intrinsic factors (e.g., genetic background, immunological status) and extrinsic variables (e.g., dietary patterns, pharmaceutical exposure, and facility-specific environmental microbiomes) [6]. At present, numerous studies have investigated the relationship between the gut microbiota and murine models of human diseases including Alzheimer's disease [7], cardiovascular disease [8], obesity [9] and diabetes [10], as well as the correlation between the diversity of intestinal microbes and metabolites [11]. Although relevant studies have analyzed and predicted the gut microbiota characteristics and functions of non-SPF and SPF mice using metagenomic sequencing technology, providing highly valuable experimental data for biomedical research, these studies still have significant deficiencies in data integrity and reproducibility [12–14]. The specific limitations are as follows: Liang et al. took non-SPF mice as the main research objects. Their sample sources covered different genetic backgrounds, suppliers and breeding laboratories, without SPF standardization control. Gut microbiota is susceptible to external factors such as bedding materials and laboratory environment, and the mouse supplier has even become the primary factor affecting the microbiota composition. Even if the subsequent breeding conditions are unified, the supplier-specific characteristics of the initial microbiota still persist, thus leading to difficulties in replicating subsequent experiments [12]. Although Zhu et al. focused on SPF-grade mice, all of their 88 newly added sequencing samples were C57BL/6 male mice, meaning the research was restricted to a single strain [13]. The SPF mice samples used by Beresford-Jones et al. were only from one institution, the Wellcome Sanger Institute [14]. Limited by this restricted subject coverage, both studies failed to fully reflect the diversity of gut microbiota in SPF mice in terms of strain and breeding institution. Therefore, to ensure the integrity of experimental data and the representativeness of experimental results, future studies should strengthen the standardized control of sample sources and expand the coverage of research subjects in terms of strains and institutions.

**Fig. 1:**
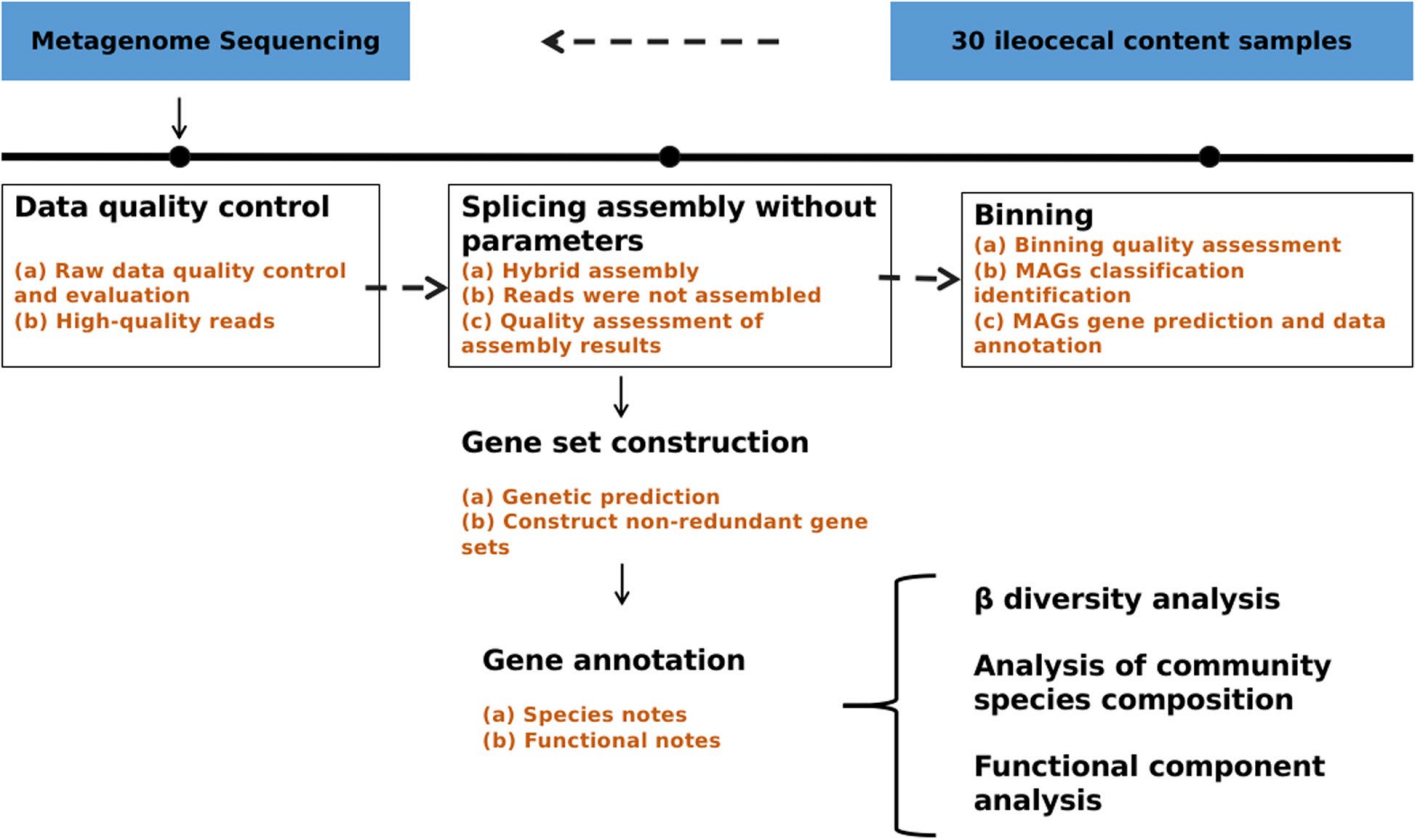
Gene Sequencing Flow Chart

Advancements in technology and the reduction in sequencing costs have made it more convenient to obtain the gut microbiota of multi-strain mice from various institutions via metagenomic sequencing [15]. In this investigation, we implemented shotgun metagenomics to systematically profile both taxonomic profiles and microbial-derived drug-resistance genes clusters in murine. Metagenomic sequencing provides profound insights and comprehensive understanding of the gut microbiota in a healthy state. This study aims to 1) identify significant taxonomic features in the two SPF mice strains from the five laboratory animal facilities, 2) determine the core microbial features specific to SPF mice of the same strain and shared by all individuals within the strain, 3) reveal the carriage status of ARGs in the gut microbiota of SPF mice. The dataset establishes a theoretical foundation for diseases treatment, and provides basic materials for the screening of experimental model animals, which plays an important role in the research and development of life science (Fig. 1). SPF mice are indispensable preclinical models in biomedical research, pivotal for investigating the gut microbiome’s role in complex disease contexts. However, comprehensive, high-resolution profiling of their gut microbiota especially ARGs, has been extremely limited. Our study addresses this gap via metagenomic sequencing of cecal contents from C57BL/6 and BALB/c SPF mice across five laboratory animal facilities. Key findings demonstrate that inter-facility variation exerts a significantly greater influence on microbial community structure and diversity than the host strain, confirming that different facilities had a greater impact. The analysis precisely delineated the ARGs carriage status, identifying 11 prevalent gene types (e.g., vanR, tet(O), mdtB), primarily hosted by four major bacterial phyla (Pseudomonadota, Bacillota, Bacteroidota and Actinomycetota), and clarification of microbiota-resistome correlations. The observed differences in ARGs resistance mechanisms across facilities link microbial phenotype to potential differences in environmental selection pressures. These results academically expand the characterization of SPF mouse gut microbiota and establish a necessary baseline for ARGs carriage profiling. Practically, they provide critical reference data for the standardization of biomedical research by mitigating experimental bias arising from inter-facility microbial variations, supporting microbial risk assessment, and ultimately enhancing the rigor of preclinical-to-clinical translation.

## Results

### Sequence analysis

The study used metagenomic sequencing data on 30 cecal content samples from two strains of SPF mice, which were obtained from five licensed laboratory animal facilities during routine regulatory inspections. The statistical table of raw data information and the statistical table of post-QC data are presented in Supplementary Table 1 and Supplementary Table 2, respectively. High-throughput sequencing of DNA samples generated a total of 141 Gbp of high-quality raw data from 30 samples, with an average data volume of 4.7 GB per sample (Supplementary Table 1). The statistical results of the gene set derived from 30 SPF mice (representing two strains and sourced from five laboratory animal facilities) are presented in Table 1.

**Table 1:** Information of the SPF grade laboratory animals

Animal number	Strains	Number	Source	Group	SRX
Ab1	BALB/c	3	SHA	A	SRX27782469
Ab2					SRX27782470
Ab3					SRX27782481
Ac1	C57BL/6	3	SHA	B	SRX27782492
Ac2					SRX27782493
Ac3					SRX27782494
Bb1	BALB/c	3	SHB	C	SRX27782495
Bb2					SRX27782496
Bb3					SRX27782497
Bc1	C57BL/6	3	SHB	D	SRX27782498
Bc2					SRX27782471
Bc3					SRX27782472
Cb1	BALB/c	3	SHC	E	SRX27782473
Cb2					SRX27782474
Cb3					SRX27782475
Cc1	C57BL/6	3	SHC	F	SRX27782476
Cc2					SRX27782477
Cc3					SRX27782478
Db1	BALB/c	3	SHD	G	SRX27782479
Db2					SRX27782480
Db3					SRX27782482
Dc1	C57BL/6	3	SHD	H	SRX27782483
Dc2					SRX27782484
Dc3					SRX27782485
Eb1	BALB/c	3	SHE	I	SRX27782486
Eb2					SRX27782487
Eb3					SRX27782488
Ec1	C57BL/6	3	SHE	J	SRX27782489
Ec2					SRX27782490
Ec3					SRX27782491

We have conducted quality assessment on the assembled genes, and the post-quality control (QC) data following metagenomic assembly are presented in Supplementary Table 3. The statistical results of the non-redundant gene set are presented in Supplementary Table 4. To avoid interference from host or non-microbial contamination, 713,334 sequences were removed, resulting in a set of 1,048,575 microbial non-redundant gene sequences retained for subsequent analysis (59.51% of the original 1,761,909 genes). The samples exhibited a GC content range of 44% to 53%, confirming the diversity and sufficient randomness of the sequencing libraries. Supplementary Fig. 1 illustrates the distribution of non-redundant gene lengths, showing distinct peaks within the 100 ~ 300 bp and 500 ~ 700 bp ranges. This bimodal distribution indicates high coverage of both shorter microbial genes and longer assembled genes. The successful retention of these longer sequences supports the robust integrity of the gene set for comprehensive functional and taxonomic analysis.

### Beta diversity analysis

**Fig. 2:**
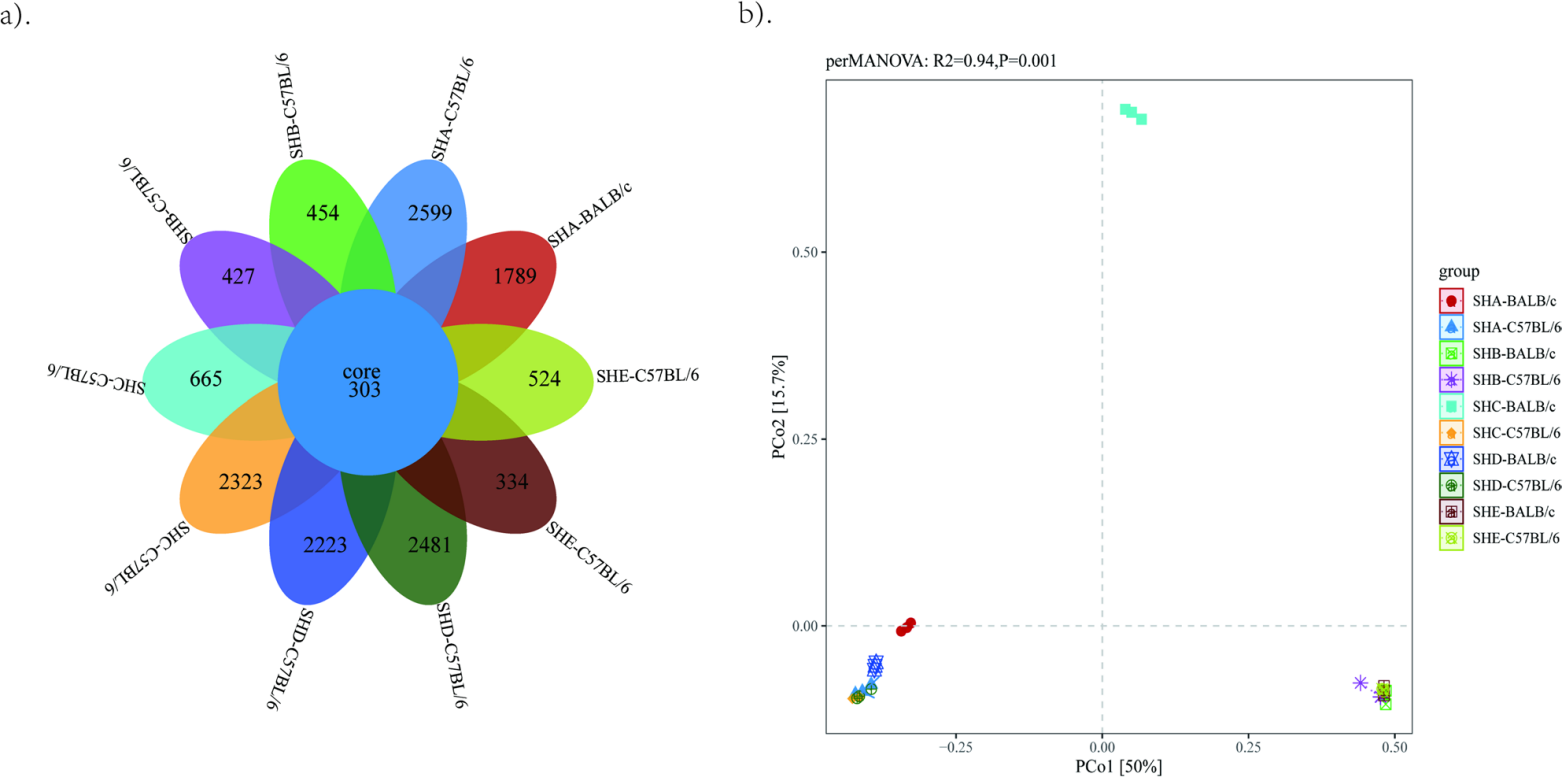
Beta diversity analysis of gut microbiota in SPF mice from five facilities. **a** Venn diagram for multi-sample comparison at the species level. Different colors represent different samples. The values in the diagram indicate the count of unique or shared taxa across samples. **b** Principal coordinate analysis (PCoA). Each shape represents a sample and each colour respresents a mouse strain from a facility

All samples together contained 303 species (Fig. 2a). C57BL/6 mice from SHA facility contained the highest species richness, while BALB/c mice from SHE facility showed the lowest number of species. PERMANOVA analysis based on Bray–Curtis distance (999 permutations) revealed a highly significant difference in beta diversity among the overall groups (R^2^ = 0.936, *p* = 0.001). Additionally, R^2^ values for all pairwise group comparisons exceeded 0.76, suggesting the presence of persistent and appreciable community structure differentiation between groups. According to beta diversity (Bray–Curtis dissimilarity) (Fig. 2b, Supplementary Fig. 2 ~ 3), groups A, B, F, G and H were close to each other, groups C, D, I and J were close to each other, but all were far from group E, indicating similar gut microbial community composition between C57BL/6 and BALB/c strains from SHA and SHD, SHB and SHE facilities.

### Species annotation analysis

**Fig. 3:**
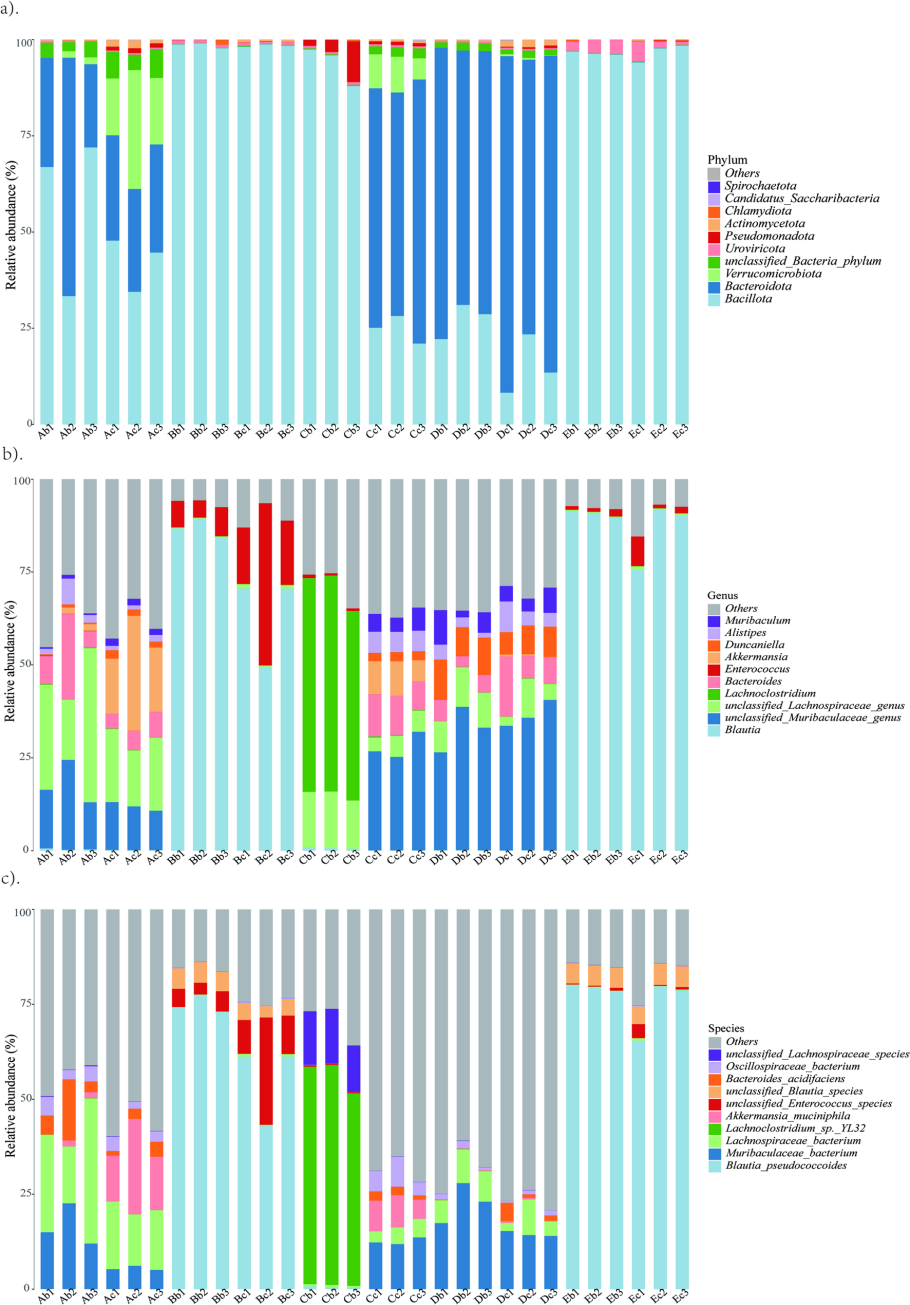
Relative abundance of gut bacteria at different levels in commonly used SPF mice. **a** Phylum level. **b** Genus level. **c** Species level. (The x-axis represents the sample IDs, and the y-axis represents the relative abundance of species. Colors correspond to the names of each species at this taxonomic level, while the widths of different color blocks indicate the relative abundance of different species. "Others" represents the total abundance of the remaining species)

Of the total classified genes, 99.62% belonged to bacteria (1,044,583 genes), with the remaining 0.38% distributed among viruses (3924 genes), fungi (34 genes), and archaea (34 genes) (Supplementary Fig. 4). *Euryarchaeota*, *Candidatus thermoplasmatota*, *Methanobrevibacter* and *Methanocorpusculum* were mainly detected in archaea. Only *Basidiomycota*, *Ascomycota*, *Puccinia* and *Pyrenophora* were detected in fungi. *Uroviricota*, *Artverviricota*, *Phixviricota* and *Hofneiviricota* were dominant phyla. Meanwhile, viral taxonomic profiling identified seven dominant genera: *Gammaretrovirus, Carjivirus*, *Intracisternal A-particles*, *Betaretrovirus*, *Baoshanvirus*, *Kayvirus* and *Pamirivirus*. Overall, the bacterial genes were annotated into 36 phyla, 846 genera and 3761 species with varying abundances. For the top three dominant phyla (3/36), the total relative abundance was 92.5%, and they were Bacillota (73.0%), Bacteroidota (16.6%) and Actinomycetota (2.9%). The total relative abundance of the top five species (5/3761) was 40.03%, and they were *Lachnospiraceae* (23.2%), *Oscillosiraceae* (7.4%), *Hungatella* sp. (3.33%), *Eubacterium* sp*.* (3.1%), *Dorea* sp. (3.0%). Furthermore, analyses (Fig. 3a, b) showed that the taxonomic profiles of different mouse strains within the same facility were largely similar, except for the SHC facility where abundances varied slightly. Conversely, the taxonomic composition of the same mouse strain across different facilities was significantly disparate.

Ten common microbial taxa, accounting for 58.44% of the relative abundance, were carried by all samples (Fig. 3c). These included key functional members such as *Akkermansia muciniphila* [16], *Bacteroides acidifacien* [17], *Blautia pseudococcoide* [18], *Lachnoclostridium sp. YL32* [19], *Lachnospiraceae bacterium* [20], *Muribaculaceae* [21], *Oscillospiraceae bacterium* [22], unclassified Blautia species [23], unclassified Enterococcus species, unclassified Lachnospiraceae species, collectively indicative of a healthy gut ecosystem. They played a key role in the gut microbiome ecosystem of healthy individuals. The bacterial taxa detected in more than 90% of the test samples were defined as the gut’s core microbiome. SHB and SHE facilities mainly contained three core micobiota, respectively *Blautia pseudococcoides*, unclassified Enterococcus species and unclassified Blautia species. There were four types core micobiota in SHD facilitiy, namely *Muribaculaceae*, *Lachnospiraceae bacterium*, *Bacteroides acidifaciens* and *Oscillospiraceae bacterium*. *Akkermansia muciniphila* was identified as an additional core taxon in the SHA facility, alongside the four species found in SHD. Notably, the core microbiome composition of the two strains within the SHC facility was highly distinct: C57BL/6 mice in SHC were similar to SHA, whereas BALB/c mice were characterized mainly by *Lachnoclostridium sp. YL32* and unclassified Lachnospiraceae species. The detailed abundance values at the phylum, genus, and species levels are provided in Supplementary Table 5.

### Function annotation analysis

**Fig. 4:**
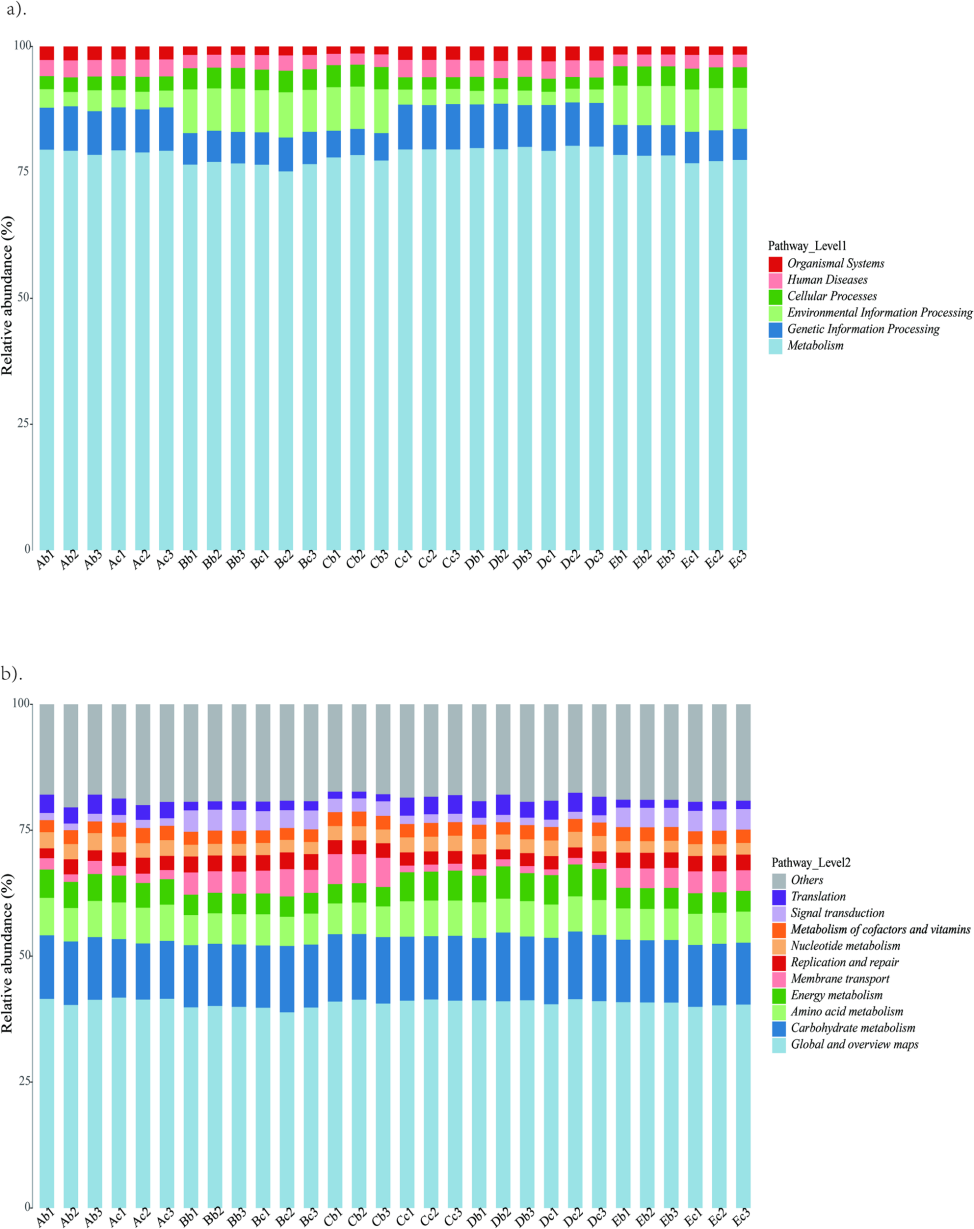
KEGG annotation of 30 samples. **a** Metabolic Annotation of Pathway Level 1 Corresponding to KO. **b** Metabolic Annotation of Pathway Level 2 Corresponding to KO. The x-axis represents 30 samples, the labels on the right indicate the full names of these functional categories, and the y-axis denotes the gene abundance contained in the corresponding major functional categories

**Fig. 5:**
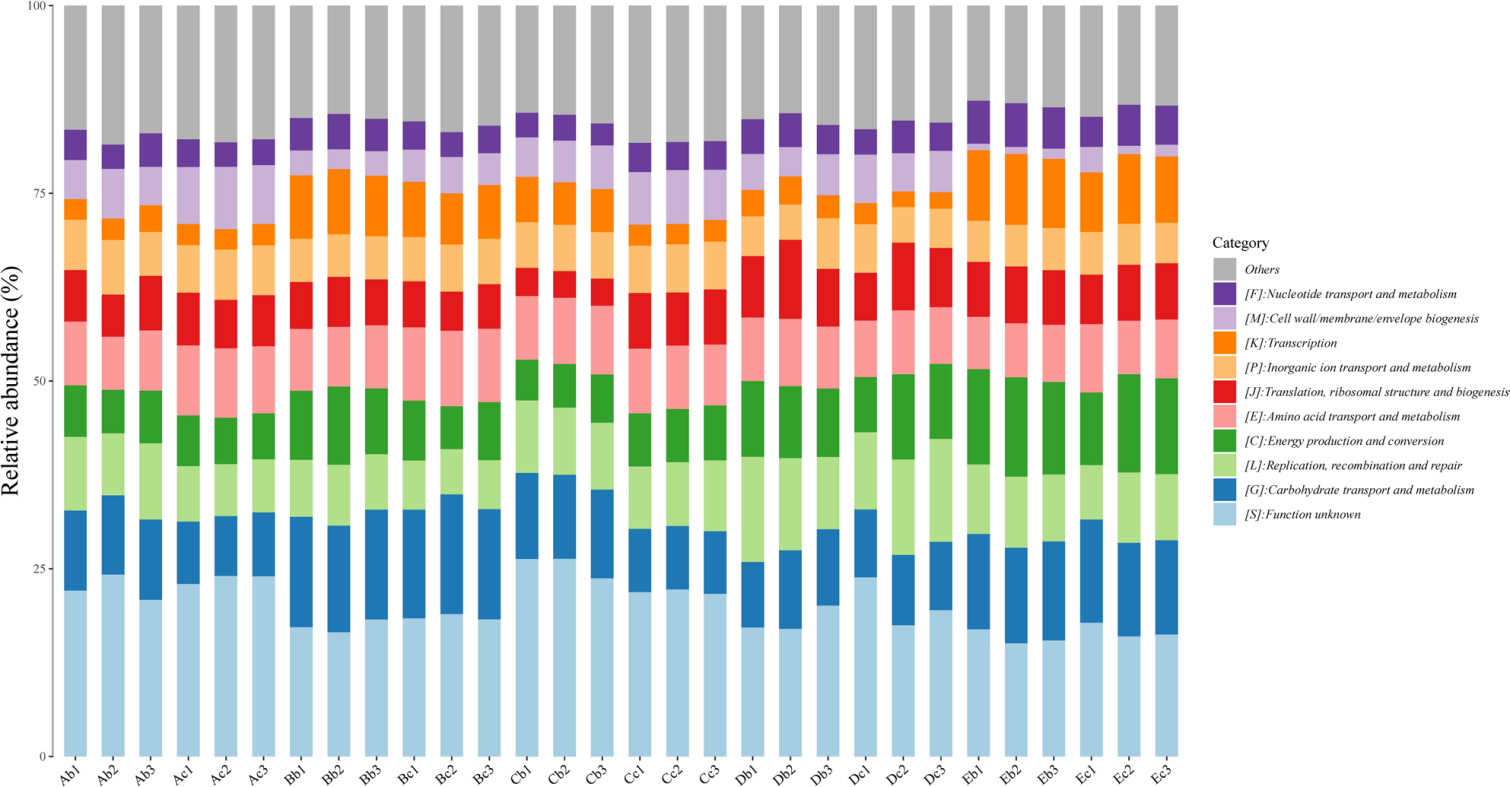
Annotation analysis of resistance genes in each Sample. **a** Results of resistance gene annotation. **b** Annotation results of resistance spectrum. The x-axis represents 30 samples, and the y-axis denotes the relative abundance of the resistance gene or spectrum

**Fig. 6:**
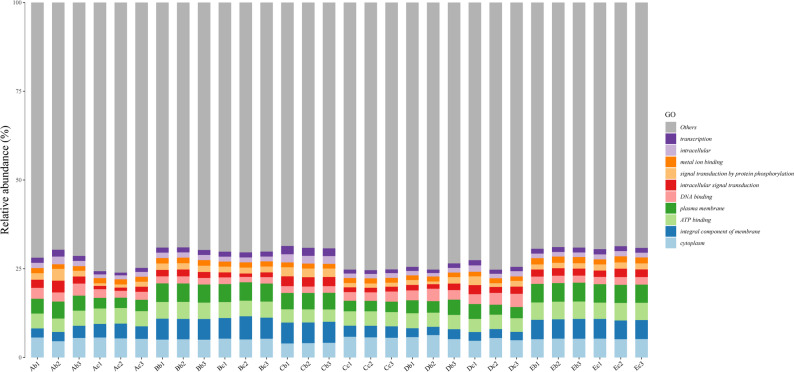
GO annotation of 30 samples. The x-axis represents 30 samples, the labels on the right indicate the full names of these functional categories, and the y-axis denotes the gene abundance contained in the corresponding major functional categories

The results of gene function classification based on Kyoto Encyclopedia of Genes and Genomes (KEGG) database showed that metabolism annotated the highest number of genes in level 1 (Fig. 4a). The level 2 classification with a large number of genes included global and overview maps, carbohydrate metabolism and amino acid metabolism (Fig. 4b). This indicated that the functional abundance of key metabolic pathways was relatively conserved across all five facilities, demonstrating low functional variation despite taxonomic differences (Fig. 4). The results of gene function classification based on eggNOG database showed that all samples contain 1,118,917 genes with unknown functions (COG category S, 22.23%), which need to be further explored. In addition, translation, ribosomal structure and biogenesis (COG category J, 10.66%), amino acid transport and metabolism (COG category E, 8.70%), carbohydrates transport and metabolism (COG category G, 9.82%), replication, recombination and repair (COG category L, 7.41%), energy production and conversion (COG category C, 6.56%), cell wall/membrane/envelope biogenesis (COG category M, 4.33%), nucleotide transport and metabolism (COG category F, 4.46%) and transcription (COG category K, 5.32%) were relatively abundant (Supplementary Fig. 5). According to Gene Ontology (GO) functional classification annotation, all samples were mainly involved in biological process, cellular component and molecular function. In biological process, translation (32,326 genes), transcription (24,691 genes) and carbohydrate metabolic process (18,461 genes) had been annotated more genes. In cellular component, cytoplasm (111,760 genes), integral component of plasma membrane (65,422 genes) and plasma membrane (46,288 genes) had been annotated more genes. In molecular function, ATP binding (142,527 genes), DNA binding (86,171 genes), metal ion binding (52,450 genes) had been annotated more genes (Supplementary Fig. 6). Overall, functional annotation revealed that the core metabolic categories were similar across different strains and facilities, though slight variations were observed in relative abundance (Figs. 4, 5 and 6).

### Annotation analysis of resistance genes

**Fig. 7:**
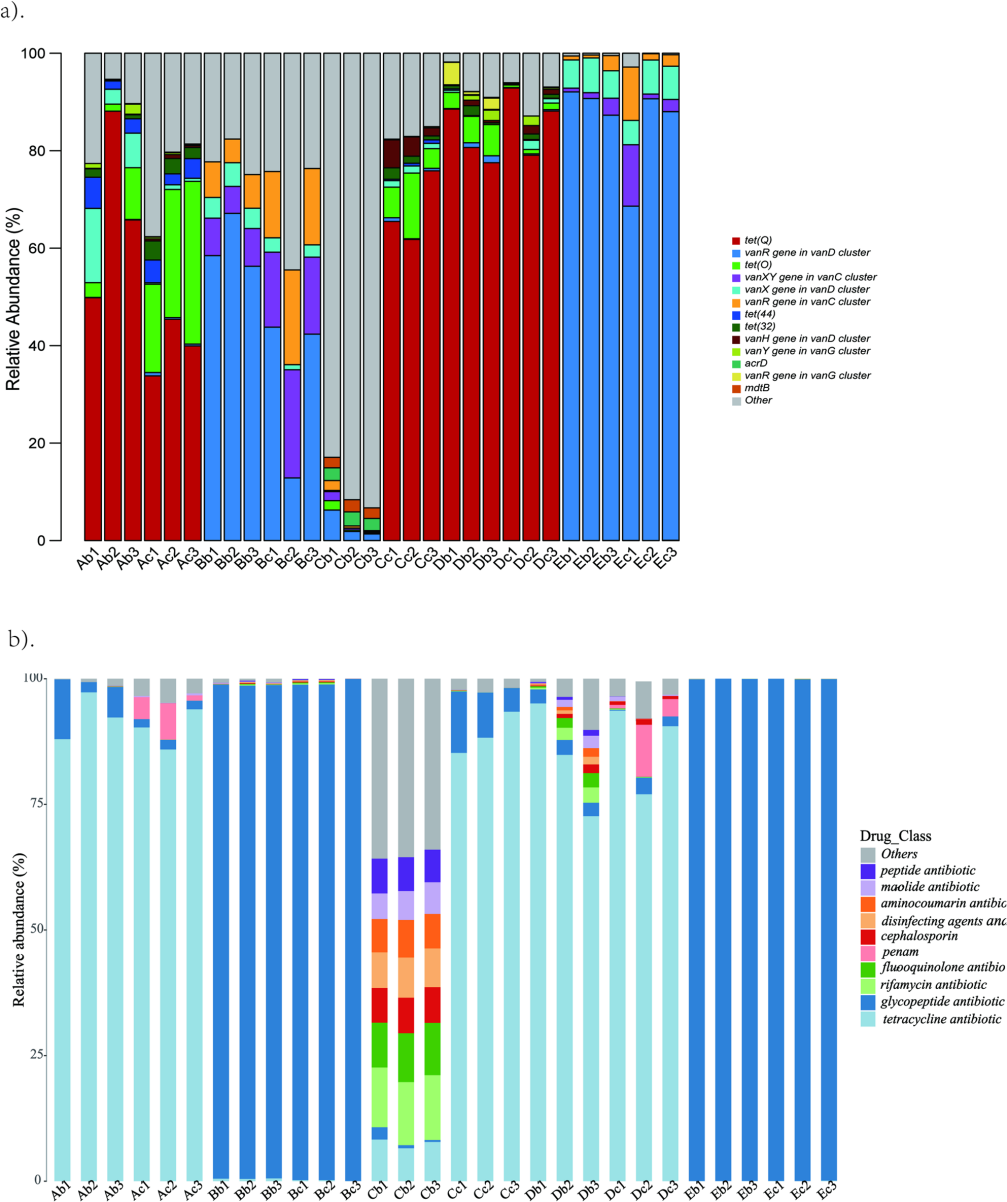
Annotation analysis of resistance genes in each Sample. **a** Results of resistance gene annotation. **b** Annotation results of resistance spectrum. The x-axis represents 30 samples, and the y-axis denotes the relative abundance of the resistance gene or spectrum

The annotation results of CARD ARGs were shown in Fig. 7a and Supplementary Fig. 7. Under stringent thresholds (E-value < 1 × 10^–5^, Score > 60), ARGs were mainly composed of glycopeptides (18.1%), tetracycline (11.3%), peptide (7.9%) and fluoroquinolone (6.0%). In addition, The 11 most prevalent ARGs were *vanR*, *vanX*, *tet*(O), *tet*(32), *tet*(Q), *tet*(44), *vanH*, *vanXY*, *vanY*, *mdtB*, *acrD*. Except for SHC, two SPF strains from the other four facilities shared similar resistance gene types and relative abundances. However, the same strain showed significant inter-institutional differences in resistance gene types, and SHC’s two strains exhibited marked differences in this aspect (Fig. 7a). Figure 7b demonstrated that mice from SHB and SHE facility develop resistance to glycopeptide antibiotics mainly through antibiotic target alteration. SHA, SHD and SHC group’s F mice develop resistance to tetracycline antibiotics mainly through antibiotic target protection, while mice SHC group’s E mice had a broader spectrum of resistance. The abundance of resistance to tetracycline, fluoroquinolones, rifamycin, penicillins, cephalosporins, disinfectants and preservatives, aminocoumarins, macrolides and peptide antibiotics was similar, while the abundance of resistance to glycopeptide antibiotics was the lowest (Fig. 5b).

Further analysis of the host sources of main resistance genes (top five) showed that *Pseudomonadota* contributed the highest proportion (57.43%), mainly *acrD*, *emrB* and *mdtB* et al. *Bacillota* (32.67%), *Bacteroidota* (7.90%), and *Actinomycetota* (1.9%) contributed smaller proportions (Supplementary Table 6).

## Discussion

In this study, our metagenomic analysis confirmed *Bacillota* and *Bacteroidota* as dominant phyla, consistent with general mammalian (including human) gut microbiota characteristics [24]. Crucially, beta diversity and taxonomic profiling demonstrated facility-of-origin dominantly exerted influence on microbial community structure, superseding C57BL/6 and BALB/c strain differences. This finding aligns with the results of Huang et al. and the principle that external environmental factors significantly shape the gut microbiota [25]. Within the SHC facility, differences between C57BL/6 and BALB/c mice may stem from segregated IVC systems with distinct environmental parameters, and variations in feed, water, management, and rearing environment [26], since different conditions alter their microbial exposure, thereby affecting intestinal flora colonization and evolution.

Although there were significant differences among different populations, the gut microbiota of healthy animals maintain conserved core features. All samples shared 10 microbial taxa accounting for 58.44% of the relative abundance, which are core to the healthy gut microbiome ecosystem. They synergistically maintain intestinal homeostasis, produce butyrate, and regulate key metabolic processes [16–21]. Crucially, core taxa profile varied by facility (e.g., three in SHB/SHE, five in SHA), demonstrating that the origin facility dictates the specific composition of even the core microbiome. Despite significant taxonomic variation across facilities, functional potential remained highly stable. KEGG and COG annotations showed core processes (metabolism, carbohydrate metabolism, and amino acid transport/metabolism) constituted the highest relative abundances, reflecting the resilience of the SPF mouse gut ecosystem. Taxa abundant across all samples, such as *Lachnospiraceae* (involved in butyrate production and barrier maintenance) and *Akkermansia* (mucin-degrading), likely contribute to this fundamental functional stability and host health [27–30].

Antimicrobial resistance genes analysis identified glycopeptide (18.1%) and tetracycline (11.3%) ARGs as main categories, dominated by van family (*vanR*, *vanX*, *vanH*, *vanXY*, *vanY*) and tet family (*tet*(O), *tet*(32), *tet*(Q), *tet*(44)). The high prevalence of the van family holds clinical relevance, as glycopeptide antibiotics (e.g., vancomycin) are the "last line of defense" in clinical treatment of multidrug-resistant Gram-positive bacterial infections, and van family genes especially *vanR* (as a regulatory gene) and *vanX*/*vanY* (as functional genes) are core elements mediating vancomycin resistance [31]. Their presence, often associated with *Bacillota*, suggests the need for continuous vigilance regarding resistance risk even in SPF mice [32]. High tetracycline ARGs abundance (11.3%) links to their "broad-spectrum and low-toxicity" application in veterinary clinics and experimental animal rearing, reflecting a positive correlation between antibiotic usage frequency and ARGs enrichment. This provides a data reference for evaluating the rationality of antibiotic use in experimental animal rearing.

Another key finding is facility-specific variation in ARGs resistance mechanisms: SHB/SHE mice mainly used "antibiotic target alteration" against glycopeptides, SHA/SHD/SHC-C57BL/6 used "antibiotic target protection" against tetracyclines, and SHC-BALB/c had a broader resistance spectrum. This divergence is strongly attributed to heterogeneity facility environmental selection pressures (e.g., disinfection methods, water treatment, or low-dose antibiotic interventions) [33], highlighting that environmental factors shape SPF mice’s resistome profile.

The distribution of host phyla for the top five resistance genes reveals distinct taxonomic specificity, providing critical insights into resistance determinant transmission and function. Pseudomonadota (57.43%) was the dominant host, with *acrD*, *emrB*, and *mdtB* as dominant subtypes, consistent with previous studies that this diverse Gram-negative phylum harbors these efflux pump genes mediating multidrug resistance, suggesting it is a core reservoir driving AMR spread [34]. In contrast, Bacillota (32.67%), Bacteroidota (7.90%), and Actinomycetota (1.9%) had lower contributions. Bacillota, predominantly Gram-positive bacteria carrying resistance genes associated with cell wall synthesis inhibition or antibiotic modification, plays a non-negligible complementary role in the resistance genes pool [35], while the lower proportions of Bacteroidota and Actinomycetota may reflect limited carriage of the analyzed resistance genes or niche-specific, low-abundance expression of their resistance determinants [36].

An objective limitation of this study is the inability to obtain complete details regarding diet formulations or detailed management protocols (such as disinfection methods, water treatment parameters, and details of low-dose antibiotic use) for all facilities, due to confidentiality agreements. This rendered it impossible to precisely quantify the specific regulatory effects of these core environmental factors on the gut microbiota structure, composition of antibiotic resistance genes (ARGs), and resistance mechanisms in mice, thereby limiting the in-depth analysis of the correlation mechanisms among "facilities—microbiota—resistome". Additionally, the samples used were only obtained from five laboratory animal facilities in Shanghai, failing to cover facilities from different regions and different management systems. Furthermore, the study focused solely on two commonly used mouse strains (C57BL/6 and BALB/c) and excluded other common SPF mouse strains (e.g., ICR, BALB/c-nu, etc.). As a result, it cannot reflect the impact of different genetic backgrounds on the characteristics of gut microbiota and resistome, lacking comprehensive representativeness of the SPF mouse population. To address the aforementioned limitations, future research can advance by 1) expanding sample geographic/management coverage, including more SPF strains, and building a standardized multi-center database to improve result representativeness, 2) strengthening key ARG functional validation and longitudinal tracking to clarify the "facilities—microbiota—resistome" correlation mechanism, 3) establishing unified data recording and risk assessment standards to support preclinical research standardization. Given the cross-sectional and observational design of this study, the observed microbiota–ARG relationships should be interpreted as associative rather than causal. Future work integrating longitudinal monitoring, host metadata, and experimental validation will be critical to disentangle causal mechanisms and to better understand the dynamics of ARG dissemination within laboratory animal microbiomes.

## Materials and methods

### Sample collection

A total of 30 cecal content samples from specific pathogen-free (SPF) mice were analyzed in this study. The samples were obtained from BALB/c and C57BL/6 mice originating from five licensed laboratory animal facilities in Shanghai (Table 1).

All samples were archived residual specimens generated during the 2023 Shanghai annual regulatory inspection, a government-mandated surveillance program conducted by authorized laboratory animal oversight institutions. The samples were provided by the Quality Supervision Station of the Basic Research Department, Shanghai Laboratory Animal Research Center, which is responsible for enforcing laboratory animal quality and health standards.

During the regulatory inspection, cecal contents were collected by the inspection authority in accordance with standard monitoring procedures and subsequently stored at − 80 °C. No additional animals were handled, sampled, or subjected to any procedures specifically for the purpose of this study, and the present work involved only the secondary analysis of pre-existing archived samples.

### DNA extraction and sequencing

DNA was extracted and quantitatively analyzed using E.Z.N.A.Soil DNA Kit (Omega Bio-Tek, M5635-02, USA) and DNA using a Qubit 4.0 (Thermo, USA). DNA samples were fragmented about 500 bp using a Covaris ultrasonic homogenizer (Covaris Inc., USA) in accordance with the S220 operating parameters. Subsequently, DNA libraries were constructed through a series of steps including end repair, adapter ligation, purification, and amplification. Finally, sequencing was performed on the DNBSEQ-T7 sequencing platform (BGI).

### Data evaluation and quality control

Fastp (version 0.36) [37] was used to perform statistics on the quality values and other information of the raw data. After removing adapter sequences from the reads, conducting global trimming and sliding window-based quality trimming, performing base correction, eliminating low-quality sequences, and removing host contamination, relatively accurate and valid data were obtained.

It could be seen phred quality score 20 (Q20) values of all samples were above 98% and phred quality score 30 (Q30) values were above 95%, indicating a high accuracy of base recognition in Supplementary Table 1.

### Metagenome assembly

First, Clean reads from multiple samples were subjected to co-assembly using megahit (version 1.2.9) [38]. Then clean reads of each individual sample were mapped to the assembled contigs via Bowtie2 (version 2.1.0) [39], followed by the retrieval of unmapped paired-end (PE) reads. Subsequently, SPAdes (version 3.13) [40] was used for co-assembly of the unmapped reads. Finally, the contigs generated from the two assembly processes were subjected to filtering to remove sequences shorter than 500 bp, followed by statistical analysis. The raw data had been submitted to NCBI and the accession number was PRJNA1226362.

### Open Reading Frame prediction and redundancy removal

The Open Reading Frame (ORF) prediction was performed by using Prodigal (version 2.60) [41] and genes with a length of 100 bp or longer were selected and translated into amino acids sequences. Redundancy removal was performed on the gene prediction results of each sample using CD-HIT (version 2.60) [42] (95.0% identity​, 90.0% coverage) to obtain a non-redundant gene set. We used Bowtie2 to align reads to human and other host genomes, and then removed the reads with high alignment similarity that were derived from host genomes or contaminants.

### Functional and ARGs annotation

Species and function annotation analysis. DIAMOND was used to compare National Center for Biotechnology Information (NCBI) Non-Redundant Protein Sequence Database (NR) (https://www.ncbi.nlm.nih.gov/protein/), Kyoto Encyclopedia of Genes and Genomes (KEGG) database (v109.0; https://www.kegg.jp/), evolutionary genealogy of genes: Non-supervised Orthologous Groups (eggNOG) database (v5.1; https://eggnogdb.embl.de/), Gene Ontology (GO) database (The Gene Ontology Consortium, 2023 release; https://geneontology.g/) and ARGs Database (CARD, McMaster University) (v3.2.7; https://card.mcmaster.ca/) to obtain gene species annotation information and functional annotation information.

Annotation analysis of ARGs. DIAMOND (version 0.8.20) [43] was used to compare the gene aggregation protein sequences with the CARD database to obtain the types and numbers of corresponding ARGs. Screening criteria: E-value < 1 × 10^–5^, Score > 60. The abundances of resistance genes and antibiotics in each sample were counted.

### Species estimation and abundance estimation

Clean reads from each sample were aligned against the non-redundant gene set using Bowtie2. Samtools (version 0.1.18) [44] was utilized to count the number of reads mapped to each gene. Considering gene length, the abundance of each gene in the samples was calculated with the formula:

1 $${G}_{k}=\frac{{r}_{k}}{{L}_{k}} \frac{1}{{\sum }_{i=1}^{n}\frac{{r}_{j}}{{L}_{i}}} 100$$

where *r* represents the number of reads mapped to the gene, and *L* denotes the length of the gene. BLASTP [45] homology alignment was performed between the protein sequences of the gene set and the Nr database using DIAMOND (version 0.8.20) [43] to obtain functional annotations and homologous species information, with the filtering criteria set as E-value < 1 × 10^–5^ and Score > 60. Meanwhile, taxonomic annotation information of the genes was retrieved from the NCBI microbial taxonomy database. The relative abundances of species were calculated at each taxonomic level, and only the top 10 species with the highest abundances were selected for constructing bar charts. Specific calculation method: After completing gene prediction for the redundancy-removed gene set, first extract the reads count of each gene and perform length correction using the formula "Ai = gene reads count ÷ gene length" (to eliminate the impact of differences in gene length). Then, sum up the Ai values of all genes to obtain the total normalized quantitative value T of the sample. Subsequently, calculate the relative abundance of each individual gene using the formula "Abundance_i = (Ai ÷ T) × 100%". Finally, based on the annotation relationship between genes and species, sum up the relative abundances of all genes belonging to the same species, and perform percentage normalization on the total abundance of all species to ultimately obtain the relative abundance of each species.

### Beta-diversity

In Beta diversity analysis, an inter-sample community distance matrix was first constructed based on the Bray–Curtis distance. Subsequently, permutational multivariate analysis of variance (PERMANOVA) was employed to test the significance of differences in community structure among groups. A total of 999 permutations were set during the analysis to ensure statistical reliability. The spatial distribution and composition differences of samples were analyzed by Venn diagram, principal coordinate analysis (PCoA), non-metric multidimensional scaling analysis (NMDS) and principal component analysis (PCA).

## Conclusions

This study utilized shotgun metagenomic sequencing to comprehensively characterize the taxonomic, functional, and antibiotic resistome profiles of C57BL/6 and BALB/c SPF mice across five facilities. We found that inter-facility differences appeared to have a stronger influence on gut microbiota composition than host strain. These findings provide reference data for the standardization of preclinical research and contribute to microbial risk assessment, thereby supporting efforts to improve the rigor and reproducibility of biomedical research.

## Supplementary Information

Supplementary Material 1: Figure S1. Density curve plot of Unique gene lengths. The horizontal axis represents the length interval, and the vertical axis represents the density, representing the density curve of the length of clustered genes.

Supplementary Material 2: Figure S2. Non-metric multidimensional scaling analysis (NMDS) for Bray-Curtis distances. Each shape represents a sample and each color represents a mouse strain from a facility.

Supplementary Material 3: Figure S3. Principal component analysis (PCA) for Bray-Curtis distances. Each shape represents a sample and each color represents a mouse strain from a facility.

Supplementary Material 4: Figure S4. Kingdom classification level abundance heatmap. Each row represents a kingdom, each column represents a sample. Color represents relative abundance, where warmer colors (e.g., red) correspond to higher relative abundance and cooler colors (e.g., blue) correspond to lower relative abundance.

Supplementary Material 5: Figure S5: Statistical Bar Chart of COG Functional Classification. The x-axis displays the abbreviations of the 25 major functional categories of COG, the labels on the right represent the full names of these functional categories, and the y-axis indicates the number of genes contained in the corresponding major functional categories.

Supplementary Material 6: Figure S6. Statistical Bar Chart of GO Functional Classification. The x-axis represents GO terms, and the y-axis represents the number of genes in each term as well as their percentage of the total number of annotated genes.

Supplementary Material 7: Figure S7. Results of resistance genes annotation. The x-axis represents resistance genes, and the y-axis denotes the number of resistance genes.

Supplementary Material 8: Table S1. Raw Data information statistics.

Supplementary Material 9: Table S2. Post-QC data statistics.

Supplementary Material 10: Table S3. Post-QC data from metagenomic assembly.

Supplementary Material 11: Table S4. Statistical results of the non-redundant gene set.

Supplementary Material 12: Table S5. Detailed abundance values at phylum, genus and species levels.

Supplementary Material 13: Table S6. Analysis of host sources of the top five major resistance genes.

## Data Availability

The raw data have been submitted to the NCBI Sequence Read Archive (SRA) with the accession number PRJNA1226362.
