# Supplementary figures and images for: Comparative metagenomic characterization of gut microbiota and antibiotic resistome in multi-facility SPF mice
Source: BMC Microbiol. 2026 Jan 15;26:170. doi: 10.1186/s12866-025-04699-6 (PMC12947448; doi:10.1186/s12866-025-04699-6)

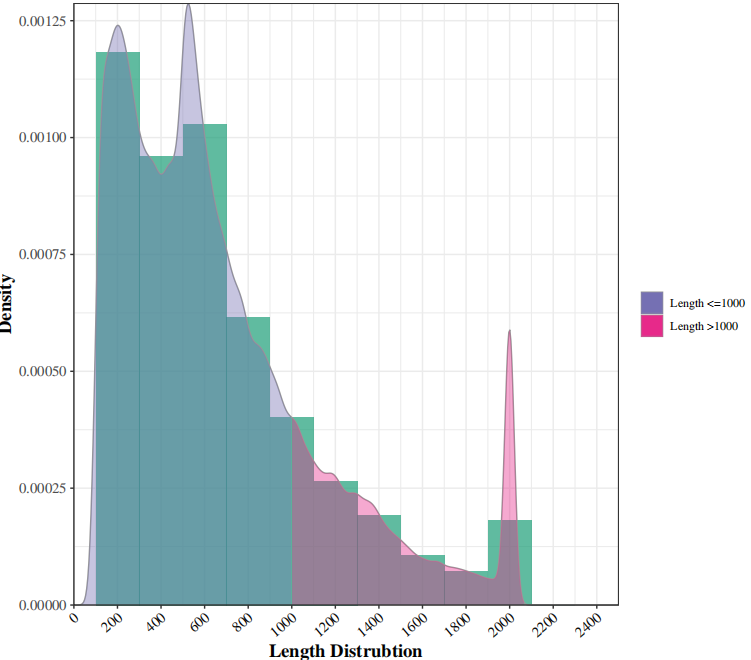

Supplement: Supplementary file 1 — Supplementary Material 1: Figure S1. Density curve plot of Unique gene lengths. The horizontal axis represents the length interval, and the vertical axis represents the density, representing the density curve of the length of clustered genes. [file 12866_2025_4699_MOESM1_ESM.tif]

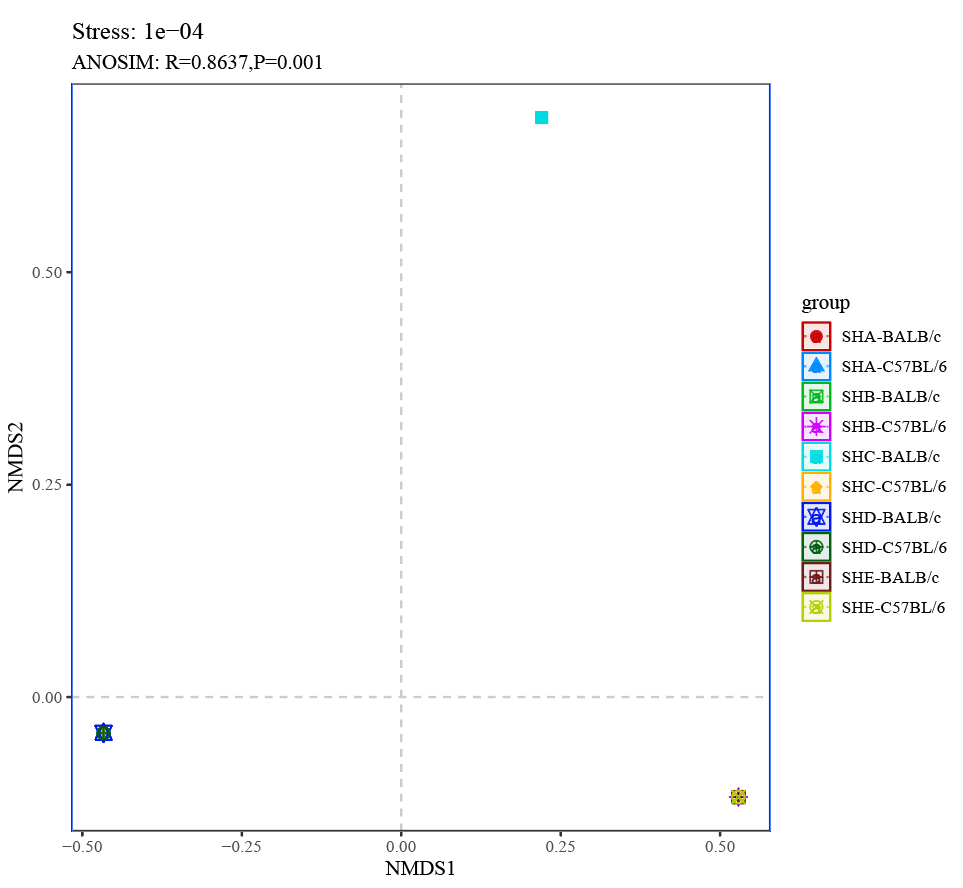

Supplement: Supplementary file 2 — Supplementary Material 2: Figure S2. Non-metric multidimensional scaling analysis (NMDS) for Bray-Curtis distances. Each shape represents a sample and each color represents a mouse strain from a facility. [file 12866_2025_4699_MOESM2_ESM.tif]

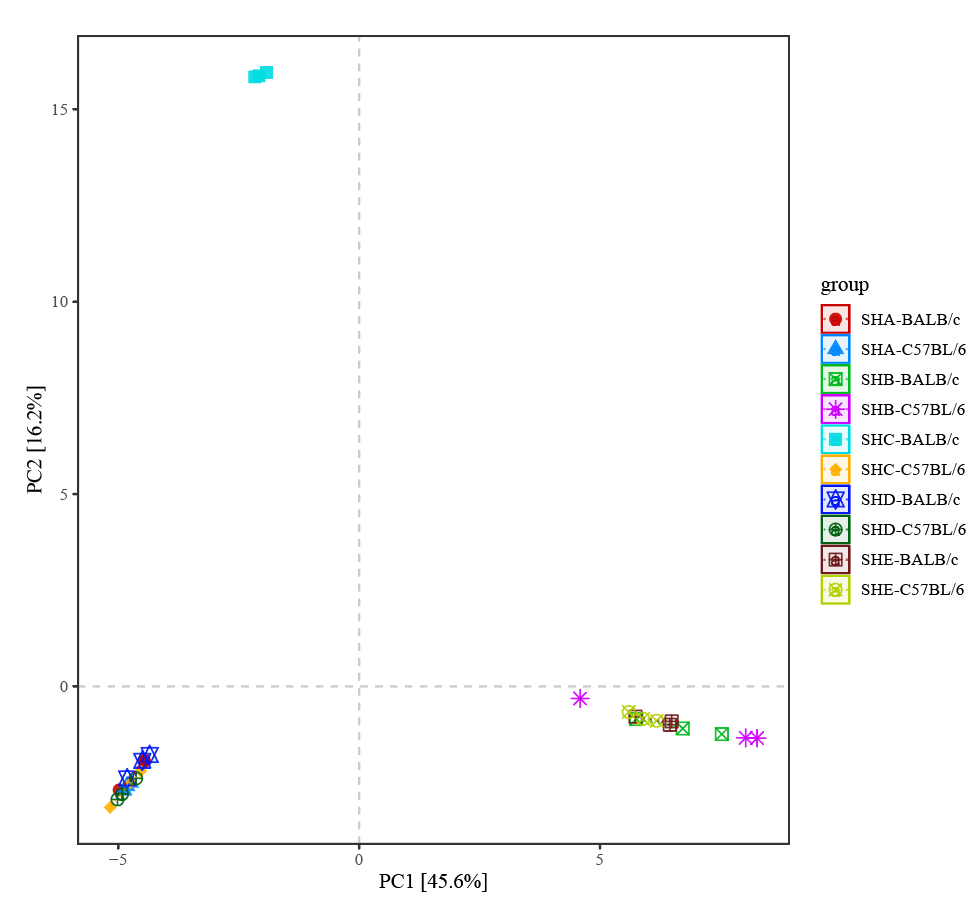

Supplement: Supplementary file 3 — Supplementary Material 3: Figure S3. Principal component analysis (PCA) for Bray-Curtis distances. Each shape represents a sample and each color represents a mouse strain from a facility. [file 12866_2025_4699_MOESM3_ESM.tif]

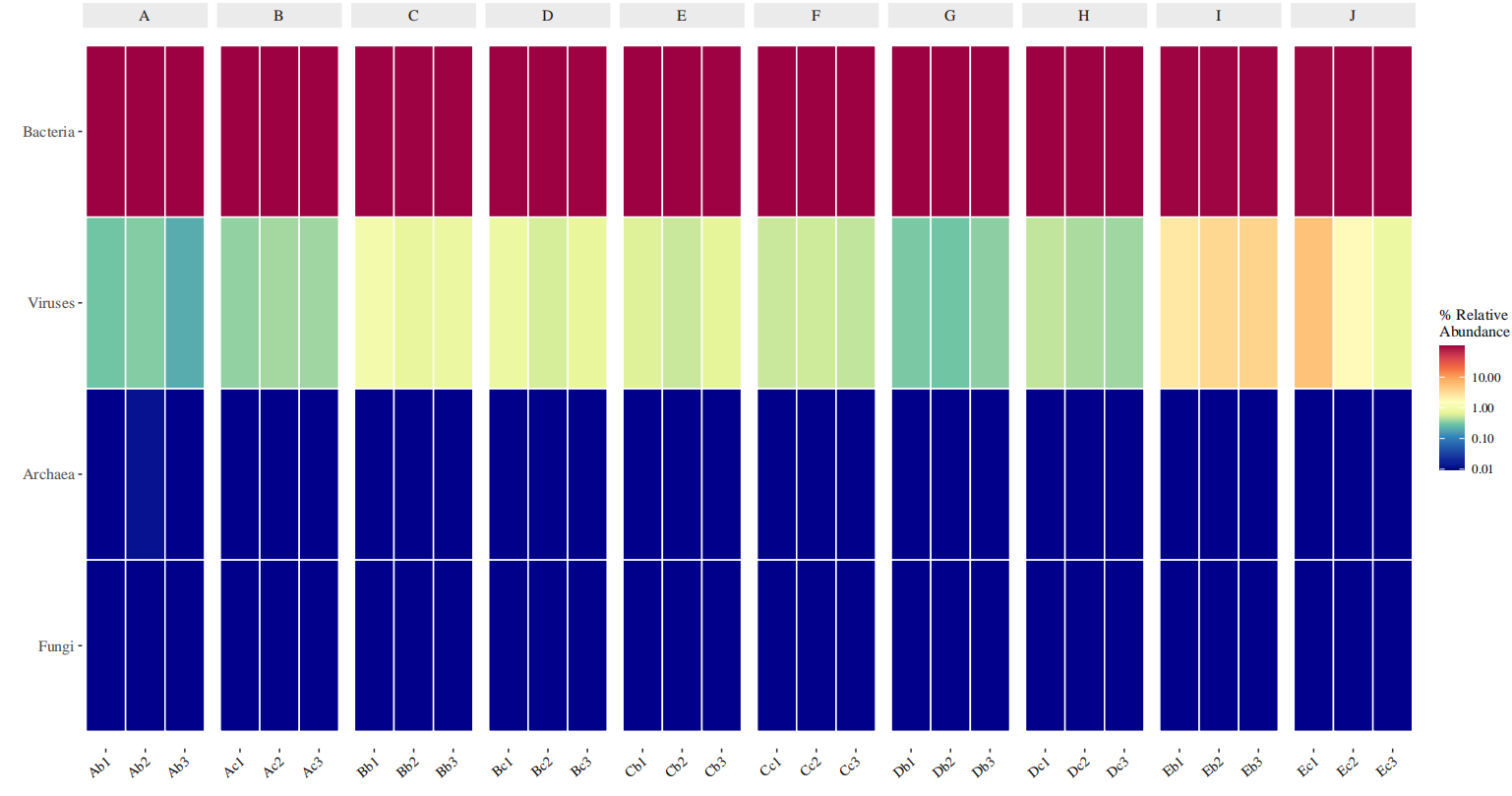

Supplement: Supplementary file 4 — Supplementary Material 4: Figure S4. Kingdom classification level abundance heatmap. Each row represents a kingdom, each column represents a sample. Color represents relative abundance, where warmer colors (e.g., red) correspond to higher relative abundance and cooler colors (e.g., blue) correspond to lower relative abundance. [file 12866_2025_4699_MOESM4_ESM.tif]

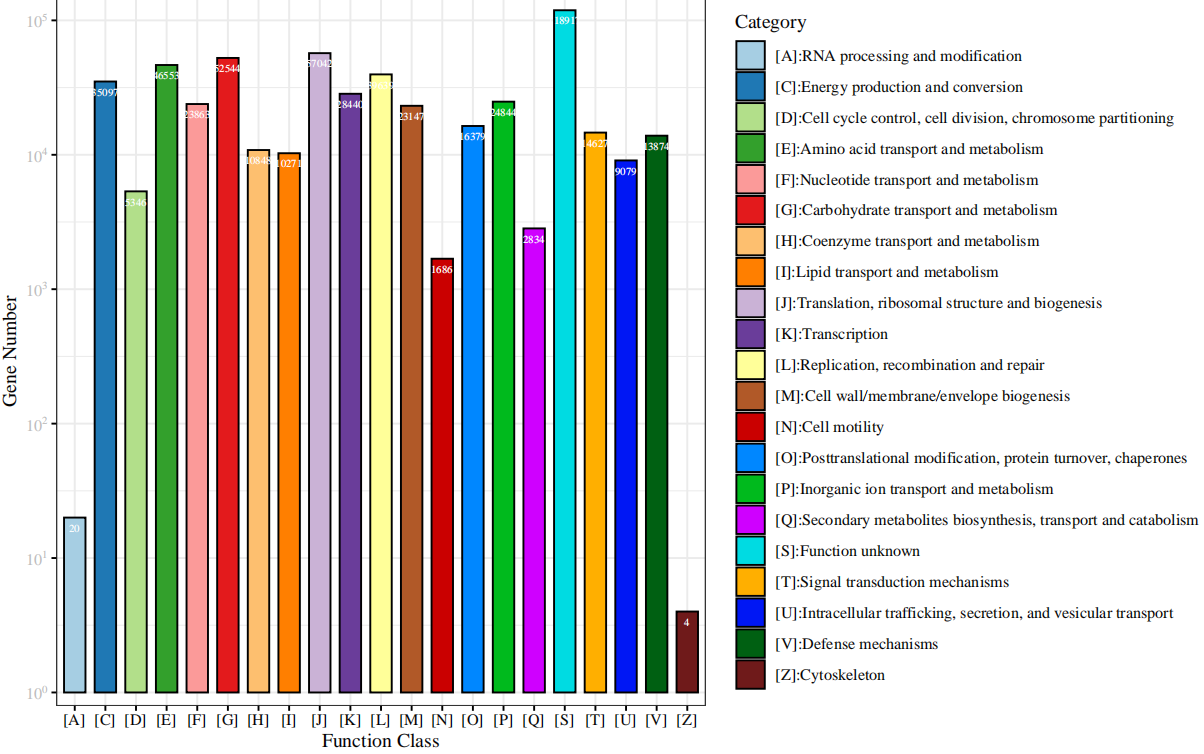

Supplement: Supplementary file 5 — Supplementary Material 5: Figure S5: Statistical Bar Chart of COG Functional Classification. The x-axis displays the abbreviations of the 25 major functional categories of COG, the labels on the right represent the full names of these functional categories, and the y-axis indicates the number of genes contained in the corresponding major functional categories. [file 12866_2025_4699_MOESM5_ESM.tif]

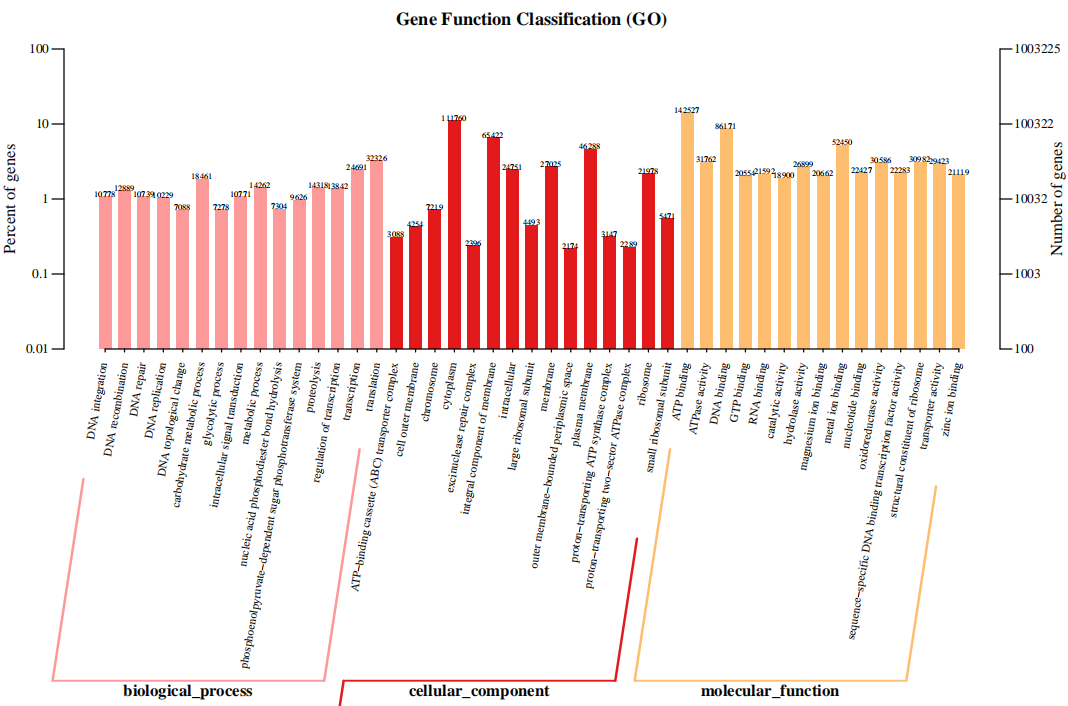

Supplement: Supplementary file 6 — Supplementary Material 6: Figure S6. Statistical Bar Chart of GO Functional Classification. The x-axis represents GO terms, and the y-axis represents the number of genes in each term as well as their percentage of the total number of annotated genes. [file 12866_2025_4699_MOESM6_ESM.tif]

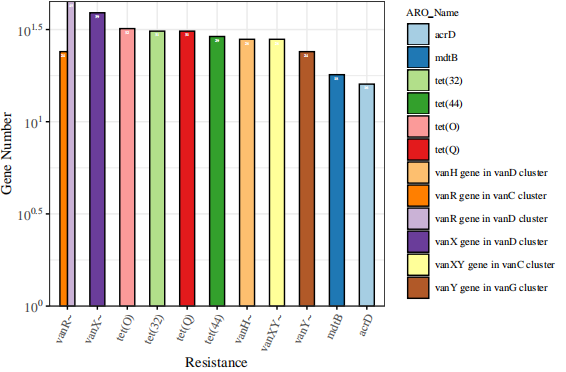

Supplement: Supplementary file 7 — Supplementary Material 7: Figure S7. Results of resistance genes annotation. The x-axis represents resistance genes, and the y-axis denotes the number of resistance genes. [file 12866_2025_4699_MOESM7_ESM.tif]
